# Baseline Red Blood Cell Distribution Width as a Predictor of Stroke Occurrence and Outcome: A Comprehensive Meta-Analysis of 31 Studies

**DOI:** 10.3389/fneur.2019.01237

**Published:** 2019-11-26

**Authors:** Si-Ying Song, Chang Hua, David Dornbors, Rui-jun Kang, Xiao-Xi Zhao, Xin Du, Wen He, Yu-chuan Ding, Ran Meng

**Affiliations:** ^1^Department of Neurology, Xuanwu Hospital, Capital Medical University, Beijing, China; ^2^Advanced Center of Stroke, Beijing Institute for Brain Disorders, Beijing, China; ^3^Department of China-America Institute of Neuroscience, Xuanwu Hospital, Capital Medical University, Beijing, China; ^4^Department of Cardiology, Anzhen Hospital, Capital Medical University, Beijing, China; ^5^Department of Neurological Surgery, Semmes-Murphey Clinic, The University of Tennessee Health Science Center, Memphis, TN, United States; ^6^Department of Ultrasonography, Tiantan Hospital, Capital Medical University, Beijing, China; ^7^Department of Neurosurgery, Wayne State University School of Medicine, Detroit, MI, United States

**Keywords:** red blood cell distribution width, stroke, risk factor, mortality, functional outcome, meta-analysis

## Abstract

**Background:** Red blood cell distribution width (RDW) may be a potential biomarker of inflammation in patients with stroke. Elevated RDW is associated with higher incidence of stroke, unfavorable functional outcome, and increased mortality, although results are inconsistent in the reported literature. This study aims to evaluate the predictive power of RDW regarding stroke occurrence and outcome.

**Methods:** A thorough literature search was conducted utilizing the PubMed Central (PMC) and EMBASE databases to identify studies up to May 2019. Data from these studies were pooled, and combined odds ratios/risk ratios (ORs/RRs) were estimated for the risk of stroke, functional outcome, and mortality. A subgroup analysis was also performed to explore heterogeneity in terms of population status, demographic factors (age, gender distribution, and country), and vascular risk factors (hypertension, diabetes mellitus, and current smoking).

**Results:** A total of 31 studies with 3,487,896 patients were included in the analysis. Elevated RDW was found to be a risk factor in ischemic stroke (OR/RR 1.528; 95% confidence interval [CI] = 1.372–1.703), whereas combined OR in subarachnoid hemorrhage (SAH) was not statistically significant (OR/RR 1.835; 95% CI = 0.888–3.792). Elevated RDW posed increased risk in populations with conventionally higher risk of stroke, such as atrial fibrillation (AF) (OR/RR 1.292; 95% CI = 1.107–1.508) and diabetes mellitus (OR/RR 2.101; 95% CI = 1.488–2.968), and in community cohorts (OR/RR 1.245; 95% CI = 1.216–1.275). In addition, higher RDW was associated with unfavorable functional outcome, either at discharge (OR/RR 1.220; 95% CI = 1.070–1.39) or at 90 days (OR/RR 1.277; 95% CI = 1.155–1.413). Higher mortality was found in patients with increased RDW (OR/RR 1.278; 95% CI = 1.221–1.337), independent of demographic factors (age, gender distribution, and country).

**Conclusions:** Baseline RDW should be integrated into clinical practice as a predictor of ischemic stroke occurrence and outcome. Future studies should also explore the dynamic change of RDW in post-stroke patients to evaluate the clinical significance of RDW and its impact on the inflammatory state of ischemic stroke.

## Introduction

Red blood cell distribution width (RDW) has served as a traditional biomarker for erythrocyte volume variability and as an indicator of erythrocyte homeostasis (1). However, the clinical significance of RDW is often overlooked and has historically been restricted to a narrow differential diagnosis, centered on anemia. Recent studies have shown that RDW elevation is seen in many human diseases, including cardiovascular diseases (2, 3), thrombosis (3, 4), and stroke (4, 5).

Inflammation has a profound impact on stroke development (6), and RDW is known to be closely associated with inflammatory responses on the basis of previous studies (1, 4, 7, 8). The relationship between RDW and stroke has begun to emerge with a large amount of evidence suggesting that elevated RDW might predict the incidence of stroke (4, 9, 10). Moreover, poor outcome in stroke is also related to a high baseline RDW level (11–13). Nevertheless, the clinical significance of RDW in stroke has not been comprehensively investigated owing to variations in sample populations and methodologies among current studies. This meta-analysis aims to evaluate the clinical value of RDW in stroke.

## Methods

### Search Strategy

This meta-analysis was registered in PROSPERO (International Prospective Register of Systematic Reviews) with the number CRD42018105318 and was conducted based on PRISMA (Preferred Reporting Items for Systematic Reviews and Meta-Analyses) guidelines (Supplementary Table 6). PubMed Central (PMC) and EMBASE databases were searched to identify studies up to May 2019. Medical subject headings and Emtree headings were used with the following keywords: “red blood cell distribution width OR RDW” and “prognosis OR prognostic OR survival OR outcome” and “stroke OR brain ischemia OR brain infarction OR cerebral infarction OR intracerebral hemorrhage OR intracranial hemorrhage.” The full search strategy is presented in Supplementary Table 1.

### Study Selection

Prospective or retrospective studies that evaluated baseline RDW level prior to any treatment in patients with a confirmed diagnosis of ischemic stroke (IS) or subarachnoid hemorrhage (SAH) were included. Studies were identified as eligible if they provided hazard ratio (HR), odds ratio (OR), or relative risk ratio (RR) with 95% confidence interval (CI) regarding the risk of stroke or clinical outcomes. Studies without RDW at baseline were excluded. Furthermore, studies were eliminated if they involved patients who had nutritional deficiencies (vitamin B12 or folic acid deficiency) or hematological diseases (primary or secondary anemia, lymphoma/leukemia, and sickle cell disease/trait) or received a blood transfusion within 2 weeks. Conference abstracts, review articles, case reports, letters, animal studies, or *in vitro* studies were not included in the analysis. If two or more studies had duplicate or overlapping data, the study with a larger sample size was used. Two reviewers (SY-S and C-H) independently performed the study selection and resolved any disagreements via discussion.

### Data Extraction

Two authors (SY-S and C-H) extracted data from all included studies, which was secondarily assessed by another author (RJ-K). Data extracted included the name of the first author, year of publication, country, study characteristics (sample size, age, and gender), clinical characteristics (population status and comorbid status), sample time, statistical methods used to define the cutoff value for RDW, and statistical sources of OR/RR (univariate or multivariate). A female-to-male ratio (F/M ratio) was introduced to precisely assess the various gender distributions among the included cohorts, which ranged from 0 to 3.2. The F/M ratio in a female-dominant subset was more than 1.2, whereas that in male-dominant cohort was <0.8. This reference interval was defined based on averaged population size in a subgroup analysis. OR/RR and 95% CI were extracted for risk of stroke/carotid atherosclerosis/thromboembolism, mortality (short or long term), and functional outcome. SPSS 19.0 was used to calculate RR and their 95% CI on the basis of data in studies if not explicitly stated in the manuscript and no response from the investigators was received after two requests. All disagreements were resolved by consensus.

### Outcomes

In studies evaluating RDW as a predictor of stroke, carotid atherosclerosis, or thromboembolism in certain cohorts, the incidences of these events were recorded. In studies assessing RDW as a prognostic factor in stroke, the modified Rankin scale (mRS) was used to measure the functional outcomes in clinical follow-up. Death was defined as mRS of 6, whereas an unfavorable outcome was identified as mRS of 3–5.

### Statistical Analyses

STATA version 14.0 (STATA, College Station, TX) was utilized in all analyses. Multivariate-adjusted OR/RR was prioritized, and univariate OR/RR was included in the meta-analysis if no multivariate-adjusted OR/RR was reported. Pooled estimates with 95% CI were derived under the Mantel–Haenszel method. Given the large sample size, OR was assumed to be a good approximation to RR, and therefore, OR and RR were pooled together and simplified to the description OR/RR. Heterogeneity was explored comprehensively through the subgroup analysis and sensitivity analyses and was assessed using the χ^2^ test and expressed as the *I*^2^ index (25% = low, 50% = medium, and 75% = high) (14). The random-effects model was performed if heterogeneity was more than 50%. Assessment of publication bias was done by visual inspection of funnel plots, combined with Begg's test and Egger's test (15, 16). Moreover, Duval and Tweede's trim-and-fill method was applied to estimate the corrected effect size after adjustment for publication bias (17). Evaluation of the risk of bias in eligible studies was under predefined criteria (18–20). *P*-values < 0.05 were considered statistically significant.

## Results

### Study Characteristics

**Figure 1:**
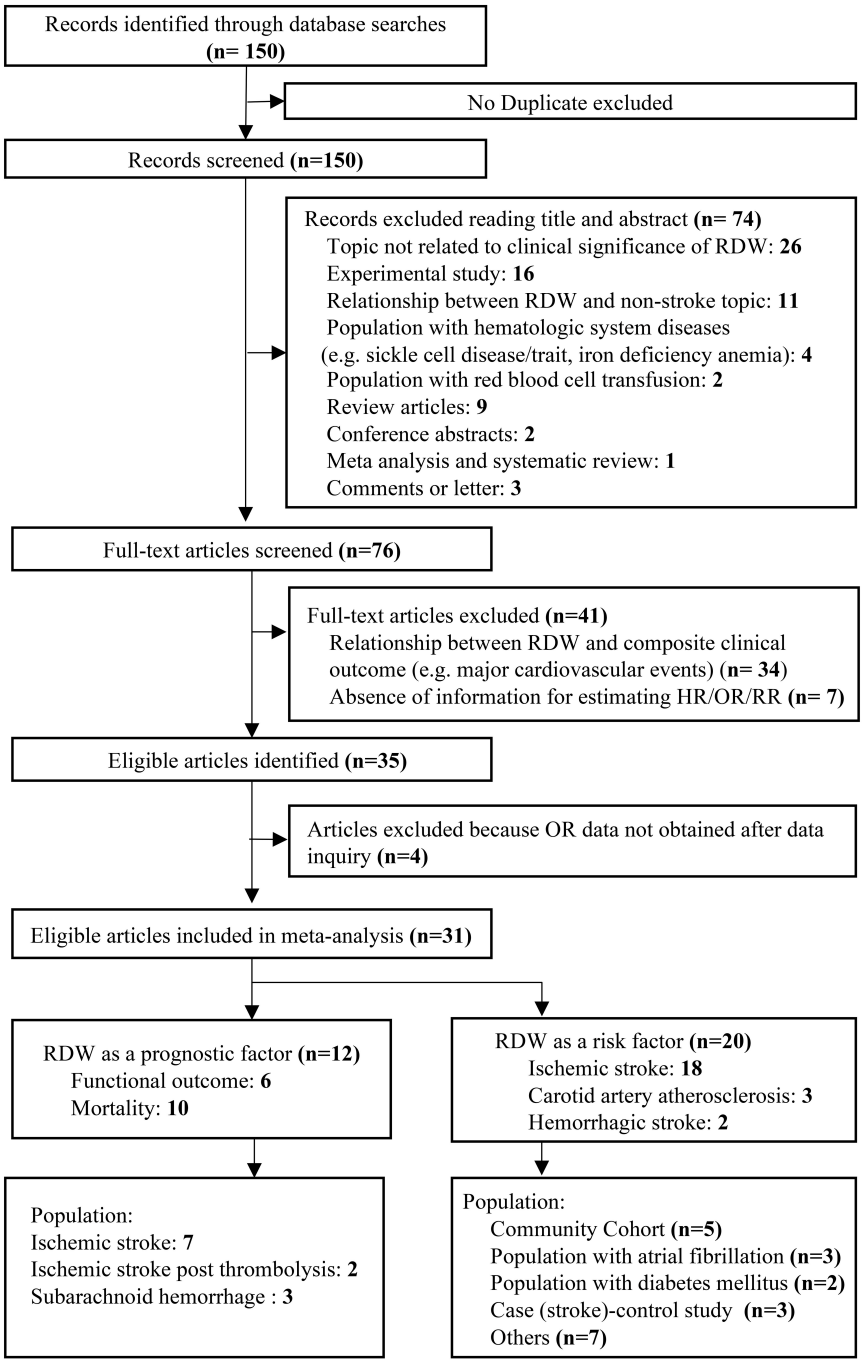
Flow diagram of the study selection process.

We identified 150 potentially relevant records and then screened them by titles and abstracts. Seventy-four studies did not meet inclusion criteria. The remaining 76 articles were retrieved for a close analysis. Ultimately, 31 studies with 3,487,896 patients were included in the analysis according to the inclusion and exclusion criteria (Figure 1). The characteristics of the included studies can be seen in Table 1 (4, 5, 9–11, 13, 21–27, 29–44).

**Table 1:** Main characteristics of 31 eligible studies included in the meta-analysis.

**References**	**Country**	**Patients number**	Age^*^	**Gender (F/M)**	**Population status**	**SAD**	**HTN**	**DM**	**Current smoking**	**Hyperlipidemia**	**Sample time^#^**	**Cutoff definition**	**Cutoff value**	**Absolute % of high RDW**	**Statistical source**
Tonelli et al. (4)	Canada	4,159	NR	565/3,546	Coronary disease	NR	42.59%	14.06%	16.10%	NR	NR	4th quartile; continuous variable	13.80%	23.25%	MV
Ani et al. (21)	USA	480	NR	252/228	IS	NR	63.40%	25.40%	22.40%	73.80%	NR	4th quartile; continuous variable	13.90%	23.80%	MV
Chen et al. (9)	China	3,226	Mean 54.7	1,692/1,534	Community cohort	NR	28.90%	12.53%	36.05%	NR	Within 24 h	4th quartile; continuous variable	13.10%	48.67%	UV
Kim et al. (22)	Korea	847	65.88 ± 12.45	340/507	IS	CAD, 16.8%	72.40%	29.40%	24.80%	21.10%	On admission	Continuous variable	Non	Non	MV
Malandrino et al. (23)	USA	2,497	NR	1,387/1,110	DM	VD, 58.7%; MI, 10.9%	77.50%	100%	20.73%	NR	NR	4th quartile	13.45%	48.46%	MV
Providência et al. (24)	Portugal	247	68.0 ± 10.5	90/157	Non-valvular AF	TIA/stroke, 15.4%;VD, 52.2%;	83.80%	22.70%	NR	NR	NR	ROC	15%	48.80%	UV
Chugh et al. (13)	USA	40	52.8 ± 10.2	30/10	SAH	CAD, 12.5%	63%	10%	67.50%	NR	Within 24 h	ROC	NR	30.00%	MV
Furer et al. (25)	Israel	522	66 ± 11	141/381	Community cohort	PVD, 22%; IHD, 42%; MI, 21%; stroke, 9%	72%	36%	43%	80%	NR	NR	14.10%	30.86%	UV
Lee et al. (26)	Korea	567	52–74	217/350	Paroxysmal AF	MI, 2.6%; PAD, 0.3%; TIA/stroke, 9.0%	40.70%	13.40%	26.60%	5.80%	NR	4th quartile; continuous variable	13.90%	27.37%	MV
Jia et al. (27)	China	392	64.8 ± 9.8	191/201	IS	CAD, 11.2%	45.40%	13.78%	12.20%	NR	NR	4th quartile	NR	NR	MV
Saliba et al. (28)	Israel	41,140	74.5 ± 13.1	21,226/19,914	AF	TIA/stroke, 21%;VD, 53.7%	78.20%	35.30%	NR	NR	Within the previous 1 year	4th quartile; continuous variable	15.00%	24.74%	MV
Söderholm et al. (29)	Sweden	26,879	45–73	16,561/10,318	Community cohort	NR	60.80%	2.90%	28.20%	NR	NR	4th quartile	NR	25.14%	MV
Vayá et al. (30)	Spain	163	43.5 ± 11.4	82/81	IS (cryptogenic subtype) vs. control	NR	NR	NR	NR	NR	NR	NR	14%	15.19%	MV
Wang et al. (31)	China	209	78 ± 8	119/90	IS	NR	77.47%	20.40%	NR	NR	Within 24 h	4th quartile	13.20%	38.28%	MV
Lappegård et al. (10)	Norway	1,152	64.0 ± 12.7	521/631	Community cohort	NR	73.50%	5.40%	35.50%	NR	NR	4th quartile	13.50%	17.18%	MV
Miller et al. (32)	USA	188	53.0 ± 13.8	42/146	Post-left ventricular assist devices vs. control	NR	NR	44.70%	NR	NR	Within 24 h	NR	18.10%	34.04%	MV
Akboga et al. (33)	Turkey	277	NR	178/99	IS (CVST subtype) vs. control	NR	NR	NR	NR	NR	Within 24 h	NR	NR	NR	UV
Al-Kindi et al. (34)	USA	3,061	61 ± 14	1,523/1,538	DM	MI, 12.25%; stroke, 10.23%	NR	100%	51.09%	NR	NR	4th quartile	13.70%	24.47%	MV
Duchnowski et al. (35)	Poland	500	62.6 ± 12.4	210/290	Post-cardiac valve surgery	CAD, 35.6%; PAD, 7.6%; MI, 10.6%; stroke, 6.8%	65.80%	100%	24.20%	NR	Within 24 h	ROC	14.10%	NR	MV
Huang et al. (44)	USA	274	59 ± 16	164/110	SAH	NR	47.06%	11.76%	NR	NR	NR	Continuous variable	Non	Non	MV
Fan et al. (11)	China	362	Median 63	146/216	IS	CAD, 12.98%	80.66%	13.81%	NR	17.40%	On admission	NR	NR	NR	UV
Siegler et al. (36)	USA	179	54 (46–65)	136/43	SAH	CAD, 7.82%; stroke, 4.47%; DVT, 1.68%	56.42%	6.09%	41.34%	NR	NR	Upper limit	14.50%	52.99%	MV
Turcato et al. (37)	Italy	316	NR	162/154	IS post-thrombolysis	MI, 12.03%	72.15%	16.77%	16.77%	33.54%	On admission	ROC; continuous variable	14.50%	21.84%	UV
Turcato et al. (38)	Italy	837	77 (68–83)	NR	IS	NR	NR	NR	NR	NR	On admission	NR	13.00%	NR	MV
Liang et al. (39)	China	108	58 ± 11	24/84	IS	MI, 10.19%; stroke, 23.15%	46.30%	18.52%	50%	NR	Within 24 h	ROC	12.20%	44.00%	MV
Lee et al. (40)	Korea	657	69.4 ± 9.8	229/428	AF	NR	48.60%	19.50%	24.00%	NR	Within the previous 3 months	ROC	13.60%	53.58%	MV
Mo et al. (41)	China	442	60.4 ± 14.3	207/235	Hemodialysis	IHD, 14.6%	42.50%	31.40%	20.00%	NR	Within the previous 6 months	4th quartile	17%	29.19%	MV
Pilling et al. (42)	USA	240,477	55.05 ± 8.1	115,811/124,666	Community cohort	NR	NR	NR	11.36%	NR	NR	4th quartile	15%	2.75%	MV
Pinho et al. (12)	Portugal	602	60.5–82	345/257	IS post-thrombolysis	CAD, 7.8%	68.40%	20.80%	NR	43.90%	On admission	4th quartile; continuous variable	Non	Non	MV
Khongkhatithum et al. (43)	Thailand	233	NR	97/136	IS vs. control	NR	NR	NR	NR	NR	NR	NR	15%	NR	UV
Tonelli et al. (5)	USA	3,156,863	NR	NR	Community cohort	NR	NR	NR	NR	NR	NR	Upper limit	15.60%	4.19%	MV

*IS, acute ischemic stroke; SAH, subarachnoid hemorrhage; CVST, cerebral venous sinus thrombosis; SAD, symptomatic atherosclerotic disease; VD, vascular disease; PVD, peripheral vascular disease; IHD, ischemic heart disease; CAD, coronary artery disease; PAD, peripheral artery disease; MI, myocardial infarction; AF, atrial fibrillation; TIA, transient ischemia attack; DVT, deep venous thrombosis; HTN, hypertension; DM, diabetes mellitus; MV, multivariable model; UV, univariate model; RDW, red blood cell distribution width; NR, not reported*.

* *Age reported as either mean ± standard deviation or median (range), if not otherwise specified*.

# *Sample time was defined as time from stroke onset to time blood sample was taken*.

Twenty studies evaluated RDW as a risk factor of stroke occurrence in different cohorts, such as community cohort (*n* = 6), atrial fibrillation (AF) (*n* = 4), and diabetes mellitus (DM) (*n* = 2). In addition, 12 studies assessed the prognostic value of RDW in stroke, including IS (*n* = 9) and SAH (*n* = 3). A large number of studies reported comorbidities within their respective cohorts. Most frequently evaluated comorbidities included DM (*n* = 25), hypertension (HTN) (*n* = 23), current smoking (*n* = 19), and systemic atherosclerosis (*n* = 16). Hyperlipidemia was only described in seven studies.

Among studies evaluating stroke prognosis, a blood sample was drawn at admission or within 24 h prior to treatment. In studies exploring RDW as a potential risk factor for stroke, only a few studies (15%) reported sample collection time, in which baseline RDW values were obtained within 1 year before enrollment. Four different methods for defining cutoff values of RDW were observed in the included studies. Quartiles of RDW distribution were used most frequently (*n* = 14), followed by continuous variables (*n* = 9), area under the receiver-operating curve (ROC) analysis (*n* = 6), and upper limit of normal RDW range (*n* = 2). The range of cutoffs of RDW was 13.8–18.1%, likely due to variable definitive methods and demographic characteristics among the cohorts, such as age, gender, and country of origin.

The majority of studies enrolled patients younger than 65 years (*n* = 14), with a balanced gender composition (*n* = 10). The number of cohorts originally from Western countries (*n* = 21) was substantially more than that of cohorts from Eastern countries (*n* = 10). More than 70% of the included studies provided results analyzed from the multivariate regression model (*n* = 24) (Supplementary Table 5). In terms of study quality, 26 studies had quality scores >7 (Supplementary Table 2).

### Association Between Red Blood Cell Distribution Width and the Risk of Stroke/Carotid Atherosclerosis

**Figure 2:**
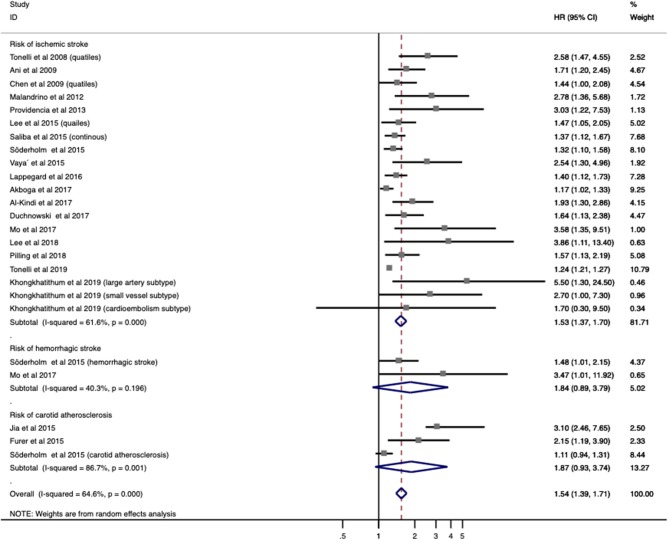
Meta-analysis of the association between RDW and risk of stroke in patients. Results are presented as individual and pooled risk ratios (RRs) with 95% confidence intervals (CIs). RDW, red blood cell distribution width.

A total of 20 studies with 3,535,653 patients provided OR/RR and 95% CI regarding the risk of IS/carotid atherosclerosis. Increased RDW was related to higher risk of combined stroke/carotid atherosclerosis (OR/RR = 1.544; 95% CI = 1.394–1.710; *I*^2^ = 64.6%; *P*_H_ < 0.001; Figure 2). By analyzing these pathologies independently, elevated RDW was found to be a risk factor in IS (OR/RR = 1.528; 95% CI = 1.372–1.703; *I*^2^ = 61.6%; *P*_H_ < 0.001; Figure 2) and carotid atherosclerosis (OR/RR = 1.869; 95% CI = 0.934–3.739; *I*^2^ = 86.7%; *P* < 0.001; Figure 2). RDW was not found to be a significant risk factor in SAH (OR/RR = 1.835; 95% CI = 0.888–3.792; *I*^2^ = 40.3%; *P*_H_ = 0.196; Figure 2).

Given the association between higher RDW and IS incidence, a further subgroup analysis was used to stratify patients by population status, demographic factors (age, gender distribution, and country), vascular risk factors (HTN, DM, and current smoking), and methodological factors (cutoff value, definition of cutoff value, and OR/RR calculation) (Table 2). Elevated RDW conferred increased risk in not only populations with conventionally higher risk of stroke, such as AF (OR/RR = 1.292; 95% CI = 1.107–1.508) and DM (OR/RR = 2.101; 95% CI = 1.488–2.968), but also non-selected community residents (OR/RR = 1.245; 95% CI = 1.216–1.275). No significant effect of high RDW was identified in either the elderly or younger populations. In terms of gender distribution, male-dominant cohorts with elevated RDW (OR/RR = 1.853; 95% CI = 1.505–2.283) were more prone to develop IS than were female-dominant cohorts (OR/RR = 1.330; 95% CI = 1.051–1.683). These results remained significant in studies performed in both Eastern and Western countries. Furthermore, the predictive value of RDW was found to be independent of vascular risk factors, such as HTN, DM, and current smoking status. Cutoff values of RDW varied among studies. Studies with cutoff values of RDW <15% were associated with worse OR/RR (OR/RR = 1.641; 95% CI = 1.453–1.855). The fourth quartile of RDW value became the most commonly used method to define a cutoff value, whereas a ROC analysis had the highest pooled OR/RR and the lowest heterogeneity among other subgroups (OR/RR = 1.890; 95% CI = 1.357–2.632, *I*^2^ = 30.0%). Both the multivariate and univariate models observed the adverse effect of RDW on IS.

**Table 2:** Subgroup analyses of the associations between RDW and risk of ischemic stroke.

**Stratified analyses**	**No. of patients**	**No. of studies**	**Model**	**Pooled HR (95% CI)**	***P*-value**	***P*_**D**_ value**	**Heterogeneity**	
							***I*^**2**^**	***P*_**H**_ value**
**Population status**						<0.001		
Community cohort	3,453,437	5	Fixed	1.245 (1.216, 1.275)	<0.001		2.9%	0.390
Atrial fibrillation	41,954	3	Random	1.292 (1.107, 1.508)	0.001		71.7%	0.007
Case (stroke)–control study	673	3	Random	2.047 (1.120, 3.740) ^*^	0.020		65.8%	0.020
Diabetes mellitus	5,558	2	Fixed	2.101 (1.488, 2.968)	<0.001		<0.001	0.381
**Demographic factors**								
**Age**						<0.001		
<65	248,146	7	Random	1.621 (1.282, 2.050)	<0.001		65.3%	0.008
≥65	69,490	5	Fixed	1.393 (1.232, 1.575)	<0.001		31.5%	0.211
**Gender distribution**						<0.001		
Female dominant	29,653	3	Random	1.330 (1.051, 1.683)	0.017		67.9%	0.045
Balanced	314,981	8	Fixed	1.521 (1.360, 1.700)	<0.001		20.0%	0.271
Male dominant	6,829	8	Fixed	1.853 (1.505, 2.283)	<0.001		19.5%	0.275
**Country**						<0.001		
Eastern	5,125	7	Fixed	1.682 (1.344, 2.104)	<0.001		31.0%	0.191
Western	3,502,735	14	Random	1.468 (1.315, 1.639)	<0.001		65.0%	0.001
**Vascular risk factors**								
**Presence of hypertension**						<0.001		
<60%	35,043	6	Fixed	1.546 (1.326, 1.803)	<0.001		45.1%	0.105
≥60%	71,743	6	Fixed	1.451 (1.292, 1.630)	<0.001		39.7%	0.141
**Presence of diabetes mellitus**						<0.001		
<20%	61,980	7	Fixed	1.446 (1.292, 1.618)	<0.001		24.4%	0.243
≥20%	47,867	6	Random	1.880 (1.434, 2.465)	<0.001		50.5%	0.073
**Presence of current smoking**						<0.001		
<25%	249,212	7	Fixed	1.851 (1.547, 2.215)	<0.001		16.8%	0.302
≥25%	59,725	5	Fixed	1.417 (1.262, 1.591)	<0.001		0.0%	0.555
**Methodological factors**								
**Cutoff value**						<0.001		
<15%	41,302	10	Fixed	1.641 (1.453, 1.855)	<0.001		22.8%	0.233
≥15%	3,439,868	8	Random	1.572 (1.260, 1.962)	<0.001		59.8%	0.015
**Definition of cutoff value**						<0.001		
4th quartile	348,920	11	Fixed	1.485 (1.357, 1.625)	<0.001		32.1%	0.143
Continuous variable	49,092	4	Fixed	1.110 (1.069, 1.153)	<0.001		46.8%	0.131
ROC curve analysis	1,404	3	Fixed	1.890 (1.357, 2.632)	<0.001		30.0%	0.240
**HR calculation**^‡^						<0.001		
Multivariate	3,503,397	13	Random	1.560 (1.365, 1.784)	<0.001		65.7%	<0.001
Univariate	4,463	7	Random	1.651 (1.218, 2.237)	0.001		58.4%	0.025

*RDW, red blood cell distribution width; HR, hazard ratio; CI, confidence interval*.

* *The result should be described as pooled OR (95% CI). All the three case–control studies (30, 33, 43) provided “OR” as results*.

‡ *HRs were extracted from multivariate Cox proportional hazards models, univariate Cox proportional hazards models or survival curve analysis*.

After a sensitivity analysis under the “one study removed” model, the pooled OR/RR was significantly affected by the exclusion of Tonelli et al. (Supplementary Table 3). Heterogeneity reduced by 5%, and the result remained statistically significant (OR/RR = 1.641; 95% CI = 1.448–1.859).

### Association Between Red Blood Cell Distribution Width and Mortality in Stroke

**Figure 3:**
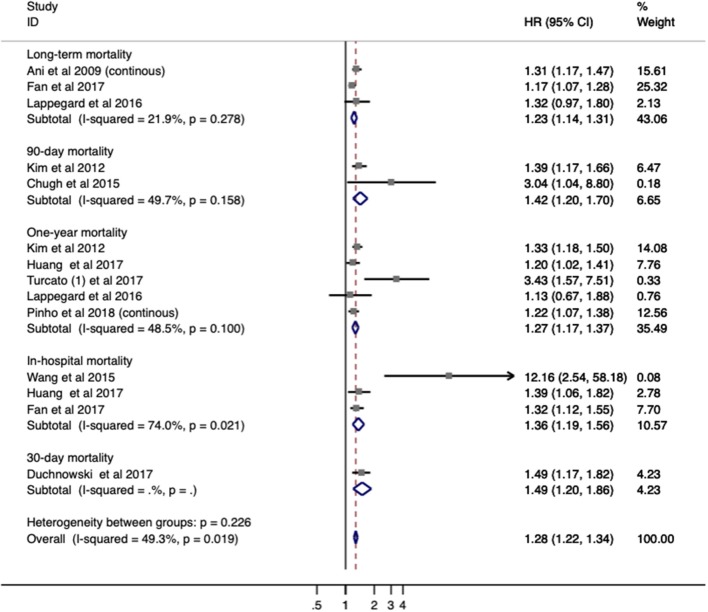
Meta-analysis of the association between RDW and mortality in patients. Results are presented as individual and pooled risk ratios (RRs) with 95% confidence intervals (CIs). RDW, red blood cell distribution width.

Ten studies with 4,782 patients were analyzed for mortality. Overall, elevated RDW was associated with increased mortality (OR/RR = 1.278; 95% CI = 1.221–1.337; *I*^2^ = 49.3%; *P*_H_ = 0.019; Figure 3). This adverse effect of higher RDW level was stronger in IS (OR/RR = 1.317; 95% CI = 1.212–1.432) than in hemorrhagic stroke (OR/RR = 1.266; 95% CI = 1.103–1.453; Table 3).

**Table 3:** Subgroup analyses of the associations between RDW and mortality in stroke.

**Stratified analyses**	**No. of patients**	**No. of studies**	**Model**	**Pooled HR (95% CI)**	***P*-value**	***P*_**D**_ value**	**Heterogeneity**	
							***I*^**2**^**	***P*_**H**_ value**
**Stroke subtype**						<0.001		
Ischemic stroke	4,468	8	Random	1.317 (1.212, 1.432)	<0.001		54.9%	0.014
Subarachnoid hemorrhage	314	2	Fixed	1.266 (1.103, 1.453)	0.018		42.3%	0.177
**Assessment time**								
**Short-term mortality**						<0.001		
In-hospital mortality	845	3	Random	1.528 (1.035, 2.257)	<0.001		74.0%	0.021
3-month mortality	887	2	Fixed	1.424 (1.196, 1.697)	<0.001		49.7%	0.158
**Long-term mortality**								
1-year mortality	3,191	5	Fixed	1.267 (1.175, 1.367)	<0.001		48.5%	0.100
Long-term mortality^*^	1,994	3	Fixed	1.226 (1.145, 1.313)	<0.001		21.9%	0.278
**Demographic factors**								
**Age**						<0.001		
<65	2,930	6	Fixed	1.238 (1.169, 1.310)	<0.001		9.5%	0.356
≥65	1,852	4	Random	1.440 (1.204, 1.721)	<0.001		70.4%	0.009
**Gender distribution**						<0.001		
Female dominant	1,125	4	Random	1.353 (1.089, 1.682)	0.006		66.0%	0.019
Balanced	1,948	3	Random	1.388 (1.088, 1.770)	<0.001		50.7%	0.108
Male dominant	1,709	3	Fixed	1.273 (1.200, 1.351)	<0.001		43.9%	0.129
**Country**						<0.001		
Eastern	1,418	3	Random	1.311 (1.150, 1.495)	<0.001		69.2%	0.011
Western	3,364	7	Fixed	1.296 (1.213, 1.385)	<0.001		35.0%	0.138
**Vascular risk factors**								
**Presence of hypertension**						<0.001		
<60%	274	1	–	–	–		–	–
≥60% and 70%	1,622	4	Fixed	1.302 (1.203, 1.409)	<0.001		39.2%	0.176
≥70%	2,886	5	Random	1.338 (1.175, 1.522)	<0.001		64.1%	0.007
**Presence of diabetes mellitus**						<0.001		
<20%	2,644	6	Fixed	1.314 (1.177, 1.466)	<0.001		47.4%	0.055
≥20%	2,138	4	Random	1.307 (1.225, 1.394)	<0.001		58.0%	0.049
**Presence of hyperlipidemia**						<0.001		
<25%	1,209	2	Fixed	1.257 (1.182, 1.337)	<0.001		39.3%	0.176
≥25%	1,398	3	Random	1.339 (1.100, 1.630)	0.004		70.6%	0.033
**Presence of current smoking**						<0.001		
<25%	2,143	4	Fixed	1.358 (1.266, 1.458)	<0.001		40.4%	0.152
≥25%	1,192	2	Fixed	1.333 (1.031, 1.724)	0.029		25.6%	0.261
**Methodological factors**								
**Sample time**^&^						<0.001		
on admission	2,127	3	Random	1.289 (1.174,1.415)	<0.001		56.4%	0.043
Within 24 h	749	3	Random	3.492 (1.301, 9.372)	0.013		71.8%	0.014
**Cutoff value**						–		
<15%	3,259	6	Random	1.908 (1.403, 2.594)	<0.001		65.7%	0.403
≥15%	274	1	–	–	–		–	–
**Definition of cutoff value**						<0.001		
4th quartile	2,443	4	Random	1.856 (1.207, 2.853)	0.005		71.3%	0.007
Continuous variable	1,929	3	Fixed	1.302 (1.221, 1.389)	<0.001		0.0%	0.637
ROC curve analysis	856	3	Random	2.207 (1.179, 4.130)	0.013		62.9%	0.067
**ORs/RRs calculation**^‡^						<0.001		
Multivariate	4,466	9	Fixed	1.270 (1.211, 1.331)	<0.001		43.1%	0.056
Univariate	1,178	3	Random	2.441 (0.974, 6.118)	0.057		76.3%	0.015

*RDW, red blood cell distribution width; HR, hazard ratio; CI, confidence interval; ROC, receiver-operating curve; ORs, odds ratios; RRs, risk ratios*.

* *Long-term mortality was defined as hazard of death due to all causes or stroke more than 1 year by the end of follow-up*.

& *Sample time was defined as time from stroke onset to time blood sample was taken*.

‡ *HRs were extracted from multivariate Cox proportional hazards models, univariate Cox proportional hazards models, or survival curve analysis*.

The subgroup analysis of RDW effect on mortality was performed regarding the aforementioned variables (Table 3). Higher RDW was more strongly correlated with short-term mortality (in-hospital mortality and 3-month mortality) than relatively long-term mortality (1-year mortality). The prognostic value of RDW was independent of all demographic factors. Cohorts with patients older than 65 years or from Eastern countries had higher pooled mortality OR/RR. In addition, increased mortality was observed in populations with a high presence of HTN (>70%) or hyperlipidemia (>25%). When stratified by methodological factors, combined OR/RR remained significant.

In the sensitivity analysis under “one study removed” model, estimated OR/RR was not significantly affected by the exclusion of any study (Supplementary Figure 3).

### Association Between Red Blood Cell Distribution Width and Functional Outcome in Ischemic Stroke

**Figure 4:**
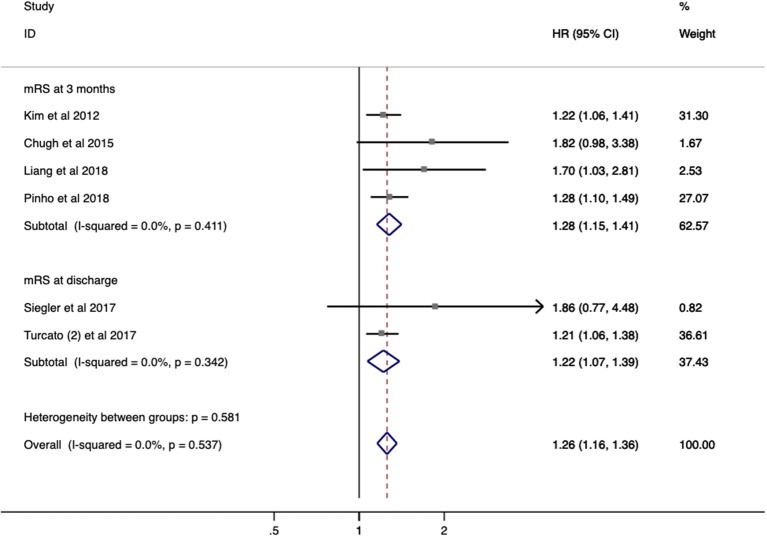
Meta-analysis of the association between RDW and modified Rankin scale (mRS) functional outcome in patients. Results are presented as individual and pooled risk ratios (RRs) with 95% confidence intervals (CIs). RDW, red blood cell distribution width.

Seven studies with 2,929 patients evaluated the relationship between RDW and functional outcome in stroke. In a pooled analysis, no significant impact on functional outcome was identified (OR/RR 1.255; 95% CI = 1.159–1.360; *I*^2^ = 0.0%; *P*_H_ = 0.537; Figure 4). Increased RDW was associated with unfavorable functional outcome both at discharge (OR/RR = 1.220; 95% CI = 1.070–1.39) and at 3-month follow-up (OR/RR = 1.277; 95% CI = 1.155–1.413). The subgroup analysis was not conducted owing to the low heterogeneity and sample size.

### Publication Bias

Evidence of publication bias in studies evaluating RDW as a risk factor (Supplementary Figure 1) and as a prognostic factor (Supplementary Figure 2) was observed for mortality in stroke by Egger's test. All combined OR/RR remained significant after the trim-and-fill method (Supplementary Table 4). Increased RDW was still associated with a higher risk of stroke and poor prognosis after stroke following adjustment for publication bias.

## Discussion

RDW is a conventional parameter, which can be easily acquired with a complete blood count (CBC) test. However, its role in reflecting inflammation has only attracted attention recently (1, 4, 7, 8). Inflammation is known to be closely related to stroke occurrence and recurrence (6, 45), and the relationship between baseline RDW and stroke has been previously assessed in other studies, albeit with variable results. This meta-analysis provides a panoramic assessment of RDW as a risk factor for stroke in various cohorts and as a negative predictor of functional outcomes. Higher RDW was found to be associated with an increased risk of IS, not only in patients with AF or DM but also in community cohorts. Further, an unfavorable functional outcome and elevated short-term mortality after stroke was identified in patients with higher baseline RDW.

Despite robust results in this analysis that higher RDW correlates with an increased risk of IS and serves as a negative prognostic factor in stroke outcome, the underlying mechanism remains unclear. Given that patients with nutritional deficiency, hematological disease, and blood transfusion were excluded from the presented analysis, the majority of patients had RDW values within the normal range, albeit on the upper limit of normal. This may suggest accelerated red blood cell destruction or, more commonly, ineffective erythropoiesis (46). In either case, there is an increased number of immature red blood cells presented in peripheral blood, resulting in elevated RDW.

It has been well-documented that inflammation is associated with the process of IS, from initial ischemia to infarction and secondary repair (6, 45). During stroke-induced inflammation, various cytokines are released and affect erythropoiesis, erythropoietin (EPO) production (47, 48), inhibition of erythroid progenitors (49), and reduction in iron release (50, 51). Further, RDW has been previously found to have a positive association with plasma inflammatory biomarkers, such as C-reactive protein (CRP) (7, 8), erythrocyte sedimentation rate (ESR) (52), and interleukin (IL)-6 (53, 54). Higher RDW, even within the normal range, may worsen the inflammatory state in stroke, leading to worse outcomes following IS. Furthermore, inflammation is known to precipitate a thrombotic state, which may underlie the increased incidence of stroke in patients with elevated baseline RDW levels (55, 56). Taken all together, elevated RDW serves as a marker for increased inflammation, whether stroke induced, leading to poor outcomes after stroke, or marking a pro-thrombotic state, resulting in increased incidence of IS.

Recent studies have shown that RDW value is influenced by demographic factors (57), including age, gender, and race. A gradual increase in RDW with age has been reported in healthy controls (1), whereas the relationship between gender and RDW is still controversial. Some studies have suggested that females have a slightly higher RDW than have males (58, 59), whereas others indicate no significant gender-based difference in RDW values (60, 61). Studies evaluating the impact of race have found that the relationship between RDW and stroke is weaker in blacks than that in whites (62). The subgroup analysis in this study was used to stratify the results by demographic factors. The results revealed that elevated RDW could predict stroke occurrence and poor survival outcome, independent of age, gender, and race. However, the clinical significance of RDW was statistically more significant in populations from Eastern countries. RDW also showed a slightly different predictive value on risk and prognosis when stratified by age and gender. Cohorts of patients younger than 65 years had a higher risk of IS but had lower mortality than do the elderly subgroup. This result may be due to the fact that the majority of studies only assessed all-cause death, rather than stroke-related death. For this reason, future studies should evaluate short-term mortality or long-term stroke-related mortality as clinical outcomes. Concerning gender, increasing incidence of IS was observed in male-dominant subsets, whereas female-dominant cohorts with higher RDW were inversely associated with survival.

Baseline RDW was evaluated as both continuous and categorical (quartiles) variables. The fourth quartile was often used to define cutoff values, ranging from 13.8 to 18.1%. However, studies utilizing a ROC analysis to identify cutoffs had more negative pooled OR/RR and lower heterogeneity. Additionally, assessing RDW as a continuous variable was more likely to have lower combined OR/RR and narrower 95% confidence intervals. The cutoff value of 14.6% has conventionally been used for anemia in the past (63). This review found that most studies chose a cutoff under 15%, which was predictive of poorer clinical outcomes. These results remained significant after a sensitivity analysis. As such, future studies could empirically use 15% as a cutoff value, conduct an individual ROC analysis, or consider RDW as a continuous variable in patients with stroke.

Several limitations should be mentioned. First, RDW could be combined with other hematological parameters, such as neutrophil-to-lymphocyte ratio (NLR), platelet-to-lymphocyte ratio (PLR), and platelet distribution width (PDW), to systematically and globally reflect the inflammatory and thrombotic state. Second, there remains controversy regarding whether an original RDW value at a certain time point or a calculated RDW value within a certain period after stroke would better predict prognosis. A calculated RDW could include mean, median, maximum, or delta RDW to encompass the dynamic nature of the inflammatory state. Future studies should follow this dynamic change of RDW in the post-stroke window, similar to that seen in Siegler et al. (36), Chugh et al. (13), and Saliba et al. (28). Third, no study has evaluated the role of RDW in predicting stroke recurrence. Long-term follow-up is suggested to study the association between RDW and stroke recurrence. Finally, there was mild heterogeneity among the included studies owing to varying populations, sample times, and methodologies to define the cutoff value of RDW. However, heterogeneity by a subgroup analysis and a sensitivity analysis (under “one study removed” model) was explored. In the subgroup analysis, heterogeneity in most of the subgroups was reduced from high-to-medium level to medium-to-low level (including subgroups of age, cutoffs of RDW, and methodologies). In the sensitivity analysis, the pooled OR/RR was significantly affected by the exclusion of Tonelli et al. as detailed above (Supplementary Table 3). Heterogeneity reduced by 5%, but the result remained statistical significant (OR/RR = 1.641; 95% CI = 1.448–1.859). To further eliminate confounding, multivariate-adjusted OR/RR was preferentially selected, and univariate OR/RR was only included in the meta-analysis if no multivariate-adjusted OR/RR existed. The majority of the included studies utilized multivariate analysis (*n* = 24), meaning that individual studies had already adjusted for potential confounding factors (gender, age, and other inflammatory markers) prior to this meta-analysis.

## Conclusions

Baseline RDW is a promising predictor of IS occurrence and outcome, independent of demographic and methodological factors. Notably, in the subgroup analysis, male-dominant cohorts with higher RDW tend to have a higher risk of stroke, and elevated RDW in the elderly population is strongly associated with mortality. Further investigation into the underlying etiology between this association between RDW and stroke risk and prognosis is certainly warranted.

## Data Availability Statement

The datasets generated for this study are available on request to the corresponding author.

## Author Contributions

RM: manuscript drafting and revision and study concept and design. S-YS: manuscript drafting and revision, study concept and design, collection, assembly, and interpretation of the data. CH and RK: collection, assembly, and interpretation of the data. RM, S-YS, CH, RK, X-XZ, XD, and WH: manuscript writing and final approval of manuscript. DD and YD deeply edited the revised version and contributed critical revision.

### Conflict of Interest

The authors declare that the research was conducted in the absence of any commercial or financial relationships that could be construed as a potential conflict of interest.
