# Supplementary material for: Baseline Red Blood Cell Distribution Width as a Predictor of Stroke Occurrence and Outcome: A Comprehensive Meta-Analysis of 31 Studies
Source: Front Neurol. 2019 Nov 26;10:1237. doi: 10.3389/fneur.2019.01237 (PMC6901990; doi:10.3389/fneur.2019.01237)
Supplement: Supplementary file 1 [file Data_Sheet_1.pdf]

Supplementary Materials

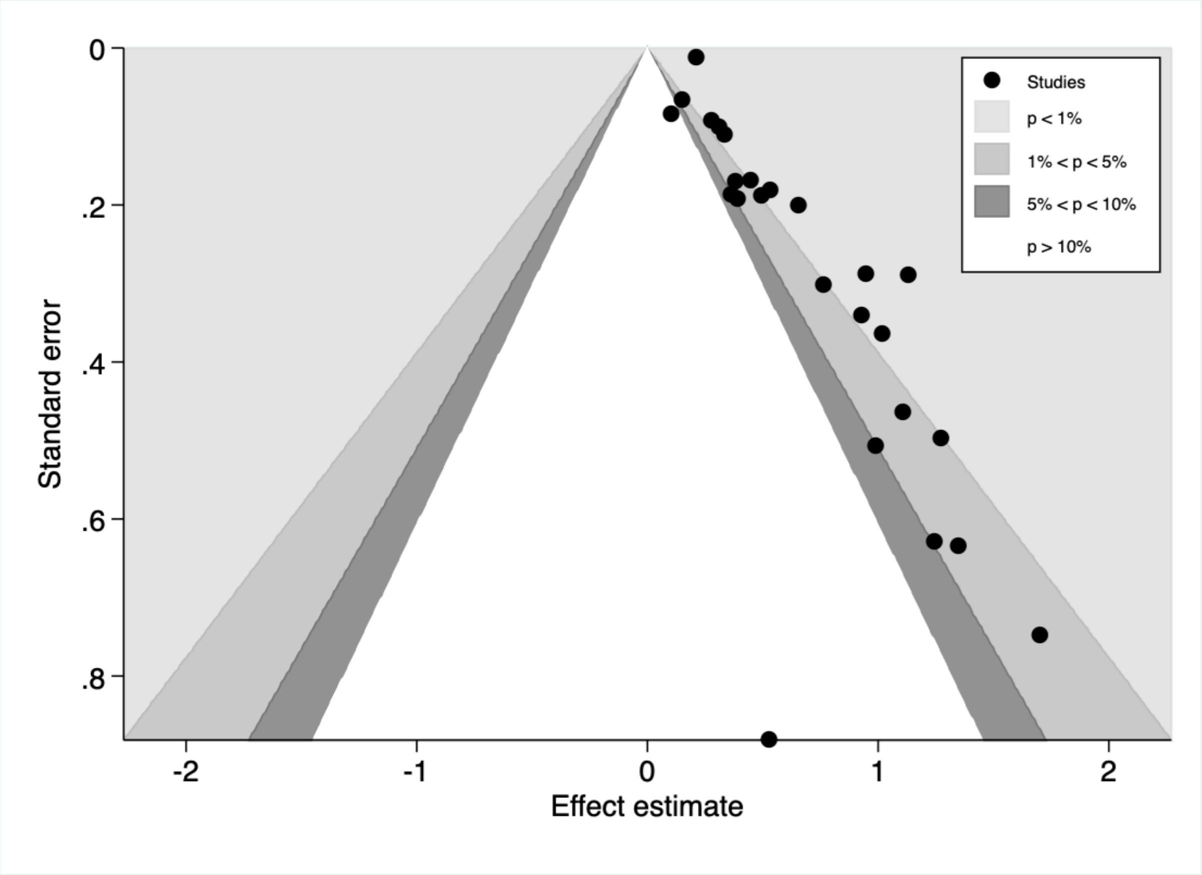

Fig. 1 Publication Bias-Funnel plot for studies evaluating RDW as risk factor. The funnel plot represents the log HR (on the X axis) against its standard error (on the Y axis) for each individual study (represented by one circle). The vertical line represents the combined effect size, with the diagonal lines representing the expected 95% confidence interval for a given standard error.

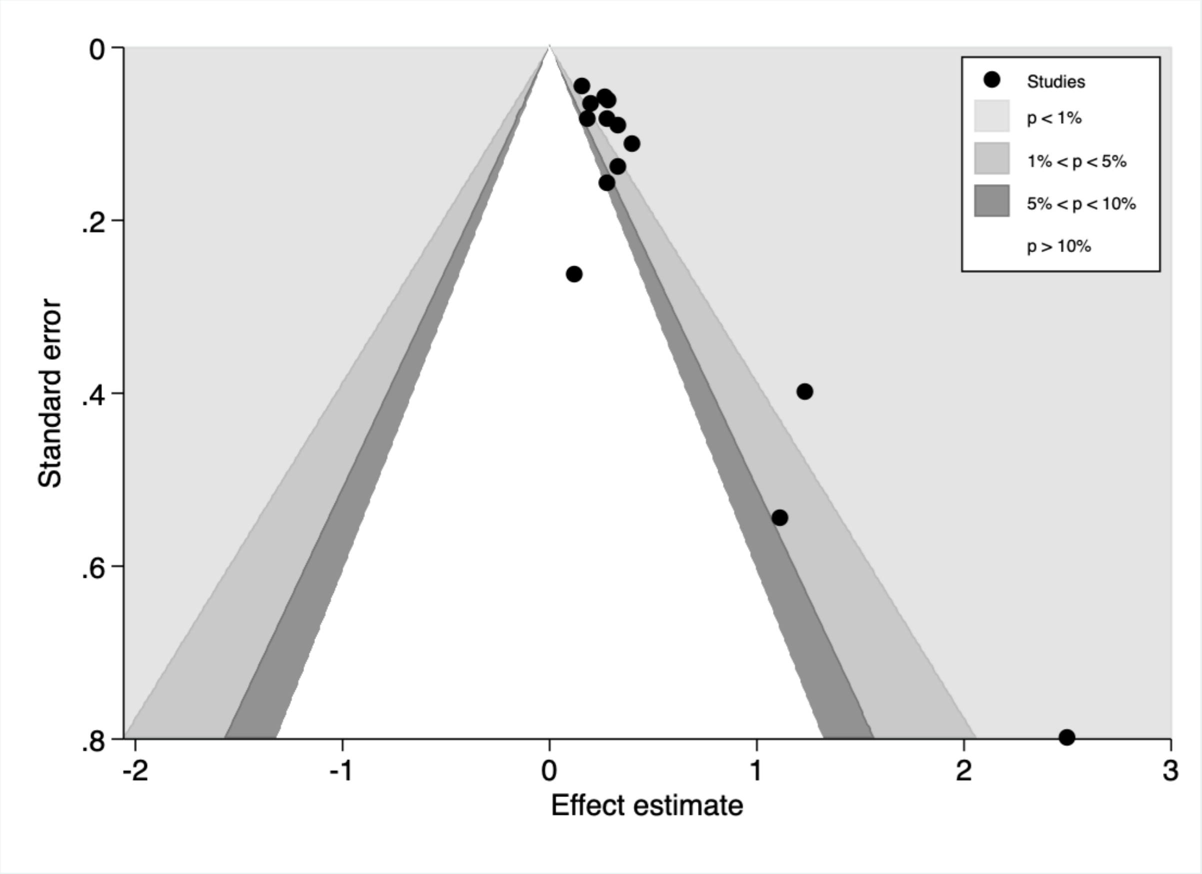

Fig. 2 Publication Bias-Funnel plot for studies evaluating RDW as prognostic factor for mortality. The funnel plot represents the log HR (on the X axis) against its standard error (on the Y axis) for each individual study (represented by one circle). The vertical line represents the combined effect size, with the diagonal lines representing the expected 95% confidence interval for a given standard error.

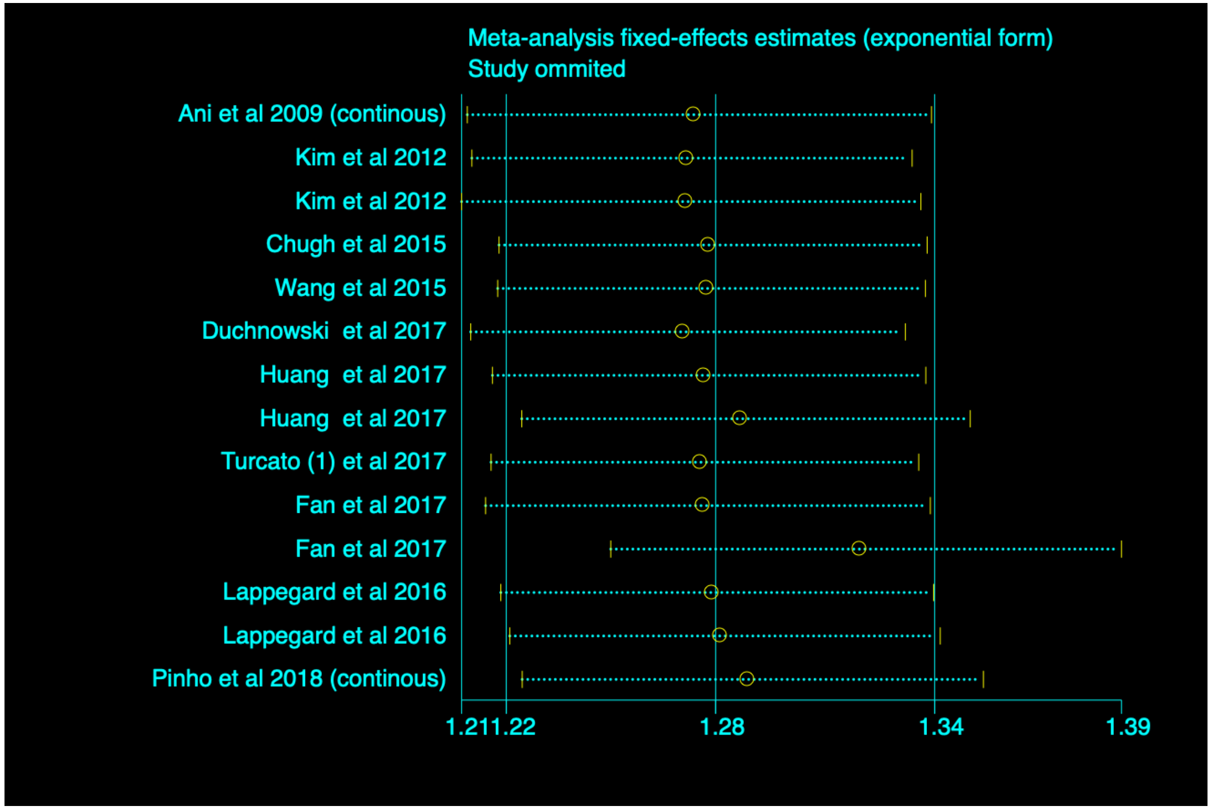

Fig.3 Sensitivity analysis using a "one-study removed" model for studies evaluating RDW as prognostic factor for mortality in stroke.

Supplementary Table. 1 Search Strategies

Search included: PUBMED, EMBASE: search date was from the inception through April 2018

1) PubMed search strategy

1.	"stroke"[Mesh]
2.	Brain Ischemia [Title/Abstract]
3.	Brain infarction[Title/Abstract]

4. cerebral infarction [Title/Abstract]
5. intracerebral hemorrhage [Title/Abstract]
6. intracranial hemorrhage [Title/Abstract]
7. 1 OR 2 OR 3 OR 4 OR 5 OR 6
8. "red blood cell distribution width" [MeSH Terms]
9. "RDW"[MeSH Terms]
10. "red blood cell distribution width"[All Fields]
11. "RDW"[All Fields]
12. 8 OR 9 OR 10 OR 11
13. "Survival"[Mesh]
14. "Mortality"[Mesh]
15. "Prognosis"[Mesh]
16. Prognos*[Title/Abstract]
17. outcome*[Title/Abstract]
18. survival[Title/Abstract]
19. mortality[Title/Abstract]
20. predict*[Title/Abstract]
21. 13 OR 14 OR 15 OR 16 OR 17 OR 18 OR 19 OR 20
22. 7 AND 12 AND 21

2) Embase search strategy

1. 'stroke'/exp
2. Brain Ischemia:ab,ti
3. Brain infarction:ab,ti
4. cerebral infarction:ab,ti
5. intracerebral hemorrhage:ab,ti
6. intracranial hemorrhage:ab,ti
7. 1 OR 2 OR 3 OR 4 OR 5 OR 6
8. red blood cell distribution width'/exp
9. red blood cell distribution width
10. red blood cell distribution width: ab, ti
11. RDW: ab, ti
12. 8 OR 9 OR 10 OR 11
13. 'prognosis'/exp
14. 'survival'/exp
15. 'mortality'/exp
16. prognos*: ab, ti
17. outcome*: ab, ti
18. survival: ab, ti
19. treatment: ab, ti
20. mortality': ab, ti
21. recurren*: ab, ti
22. predict*: ab, ti
23. 13 OR 14 OR 15 OR 16 OR 17 OR 18 OR 19 OR 20 OR 21 OR 22
24. 7 AND 12 AND 23

Supplementary Table. 2 Methodological characteristics of included studies and quality score.

No.	Authors (Ref.) *	Representativeness of population	Non-exposed cohort	Ascertainment of exposure	Outcome present at start of study	Appropriate confounding measurement and account	Sufficient measurement of outcomes	Completeness of follow-up
1	Tonelli et al 2008	2	0	0	2	2	2	1
2	Ani et al 2009	2	0	0	2	2	2	1
3	Chen et al 2009	1	0	0	2	2	2	1
4	Kim et al 2012	2	0	0	2	2	2	1
5	Malandrino et al 2012	1	0	0	2	2	2	1
6	Providencia et al 2013	2	0	0	2	2	2	1
7	Chugh et al 2015	2	0	0	2	2	1	1
8	Furer et al 2015	2	0	0	2	2	1	1
9	Lee et al 2015	2	0	0	2	2	2	1
10	Jia et al 2015	2	0	0	2	2	1	1
11	Saliba et al 2015	1	0	0	2	2	2	1
12	Söderholm et al 2015	2	0	0	2	2	2	1
13	Vayá et al 2015	1	1	0	2	2	1	1
14	Wang et al 2015	2	0	0	2	2	2	1
15	Lappegard et al 2016	2	0	0	2	2	2	1
16	Miller et al 2016	1	1	0	2	2	1	1
17	Akboga et al 2017	0	1	0	2	2	0	1
18	Al-Kindi et al 2017	1	0	0	2	2	2	1
19	Duchnowski et al 2017	2	0	0	2	2	2	1
20	Huang et al 2017	1	0	0	2	2	2	1
21	Fan et al 2017	1	0	0	2	2	0	1
22	Siegler et al 2017	2	0	0	2	2	2	1

23	Turcato (1) et al 2017	2	0	0	2	2	2	1
24	Turcato (2) et al 2017	0	0	0	2	2	1	1
25	Liang et al 2018	2	0	0	2	2	2	1
26	Lee et al 2018	2	0	0	2	2	2	1
27	Mo et al 2017	2	0	0	2	2	2	1
28	Pilling et al 2018	1	0	0	2	2	2	1
29	Pinho et al 2018	2	0	0	2	2	2	1
30	Khongkhatithum et al 2019	0	1	0	2	2	1	1
31	Tonelli et al 2019	0	0	0	2	2	2	1

Adequate assessment included 1) representativeness of population: “source population clearly defined” and “study population described” or “study population represents source population or population of interest”; 2) completeness of follow-up: “completeness of follow-up adequate”; 3) non exposed cohort: Drawn from the same community as the exposed cohort; 4) sufficient measurement of outcomes: “outcome measured appropriately”; 5) appropriate confounding measurement and account: “confounders defined and measured” and “confounding accounted for”; and 6) outcome of interest was not present at start of study

*References as described in manuscript

Supplementary Table. 3 Sensitivity analysis using a "one-study removed" model for studies evaluating RDW as risk factor of stroke

Sensitivity analysis	Heterogeneity test (I^2)	Pooled HR (95%CI)
All studies	64.6%	1.544 (1.394, 1.710)
Excluding Tonelli et al 2019	59.9%	1.641 (1.448, 1.859)

Supplementary Table. 4 Publication bias assessment with different tests for mortality and risk factor.

Publication bias	Begg's *P* value	Egger's *P* value	T&F(Fill) method analysis		Model
			Before	After	
Risk factor subset	0.002	< 0.001	1.544 (1.394, 1.710)	1.300 (1.167, 1.447)	random
Mortality subset	0.021	0.002	1.278 (1.221, 1.337)	1.260 (1.206, 1.317)	fixed

Abbreviations: CI= confidence interval; Fill=number of studies added by trim and fill method; het= heterogeneity; HR=hazard ratio; T&F=result of trimmed and filled analysis, using assumption of random effects.

Supplementary Table. 5 Confounding variables in multivariate/univariate regression model in 31 eligible studies included in the meta-analysis.

No.	Authors (Ref.) *	Outcome	Outcome source	Confounding variables
1	Tonelli et al 2008	Risk of IS	MV	Age, sex, race
2	Ani et al 2009	Risk of IS	UV	
		Prognosis-mortality	MV	Age, sex, MI, DM, Smoking, WBC, Hct, RDW
3	Chen et al 2009	Risk of IS	UV	
4	Kim et al 2012	Prognosis-mortality &functional outcome	MV	Age, sex

5	Malandrino et al 2012	Risk of IS	MV	Age, sex, race, education, smoking, hypertension, BMI, total cholesterol levels, CRP, Hb, MCV, ALB, iron deficiency, vitamin B12 deficiency, folate deficiency, HbA1c, DM
6	Providencia et al 2013	Risk of IS	UV	
7	Chugh et al 2015	Prognosis-mortality &functional outcome	MV	CBC parameters, CRP, ESR, D-dimer, fibrin, RDW
8	Furer et al 2015			
9	Lee et al 2015		MV	Age, sex, hypertension, DM, MI, HF, stroke/TIA, gastrectomy, and malignancy, CHA2DS2-VASc score, Hb, hs-CRP, creatinine clearance.
10	Jia et al 2015	risk of carotid artery atherosclerosis	MV	Smoking, Hypertension, Triglyceride, Serum uric acid, RDW, BUN
11	Saliba et al 2015	Risk of IS	MV	Age, HF, hypertension, DM, previous stroke
12	Söderholm et al 2015	Risk of IS	MV	Systolic and diastolic blood pressure, blood pressure medication, smoking, DM, alcohol intake, waist circumference, low physical activity, lipid lowering medication, WBC, AF, HF
		Risk of SAH	MV	
		Risk of carotid artery atherosclerosis		Age, RDW
13	Vaya´ et al 2015	risk of Cryptogenic Stroke	MV	Age, sex, fibrinogen, Leukocytes, Total cholesterol, BMI, Anemia
14	Wang et al 2015	Prognosis-mortality	MV	RDW, NIHSS score
15	Lappegard et al 2016	Risk of IS	MV	Age, sex

		Prognosis-mortality	MV	Age, sex, BMI, smoking, Hb, WBC, PLT, hypertension, cholesterol, triglycerides, DM, RBC, time from baseline measurement to incident stroke
16	Miller et al 2016			
17	Akboga et al 2017	Risk of IS	UV?	Hemoglobin, RDW, MPV, PLR, NLR, FPG
18	Al-Kindi et al 2017	Risk of IS	MV	age, gender, race, hemoglobin, SBP, smoking, cholesterol, and insulin use
19	Duchnowski et al 2017	Risk of IS	MV	Hb, RDW
		Prognosis-mortality	MV	Creatinine, RDW
20	Huang et al 2017	Prognosis-mortality	MV	RDW, NLR, simplified acute physiology score
21	Fan et al 2017	Prognosis-mortality	MV	Age, sex, WBC, eosinophil, monocyte, NIHSS, hypertension, DM, hyperlipidemia, CAD
22	Siegler et al 2017	Prognosis-mortality	MV	Age, sex, Hunt–Hess grade, WBC, Hb, Venous thromboembolism, stroke, RDW
23	Turcato (1) et al 2017	Prognosis-mortality	UV	
24	Turcato (2) et al 2017	Prognosis-functional outcome	MV	Age, RDW, NIHSS category, thrombolysis treatment
25	Liang et al 2018	Prognosis-functional outcome	MV	on admission NIHSS score, RDW at baseline, Glucose at baseline
26	Lee et al 2018	Risk of IS	MV	Age, sex, hypertension, DM, dyslipidemia, smoking, MI, HF, stroke/TIA, CHA2DS2-VASc score, anticoagulants, Hb, RDW
27	Mo et al 2017	Risk of IS	MV	Charlson Comorbidity Score, ALB, Atrial fibrillation, RDW
		Risk of SAH		Hypertension, Albumin, RDW

28	Pilling et al 2018	Risk of IS	MV	Age, sex, smoking status, educational attainment, Hb, MCV, RDW
29	Pinho et al 2018	Prognosis-mortality	MV	Age, sex, race, CBC parameters, early post-stroke clinical status (NIHSS 24 h after thrombolysis, symptomatic intracranial hemorrhage, early post-stroke infection), RDW
		Prognosis-functional outcome	UV	
30	Khongkhatithum et al 2019	Risk of IS	UV	
31	Tonelli et al 2019	Risk of IS/TIA	MV	Age, sex, race, morbidities, Hb, WBC, eGFR

DM = Diabetes mellitus**,** MI = myocardial infarction, CAD=coronary artery disease, HF= heart failure, TIA=transient ischemic stroke CRP = C-reactive protein, hs-CRP=high-sensitivity C-reactive protein, BUN = Blood urea nitrogen, Hct = Hematocrit, Hb=Hemoglobin, ALB=albumin, eGFR = estimated glomerular filtration rate

Supplementary Table. 6 PRISMA checklist

Section/topic	#	Checklist item	Reported on page #
**TITLE**			
Title	1	Identify the report as a systematic review, meta-analysis, or both.	Page 1
**ABSTRACT**			
Structured summary	2	Provide a structured summary including, as applicable: background; objectives; data sources; study eligibility criteria, participants, and interventions; study appraisal and synthesis methods; results; limitations; conclusions and implications of key findings; systematic review registration number.	Page 2
**INTRODUCTION**			
Rationale	3	Describe the rationale for the review in the context of what is already known.	Page 4
Objectives	4	Provide an explicit statement of questions being addressed with reference to participants, interventions, comparisons, outcomes, and study design (PICOS).	Page 4
**METHODS**			
Protocol and registration	5	Indicate if a review protocol exists, if and where it can be accessed (e.g., Web address), and, if available, provide registration information including registration number.	Page 4
Eligibility criteria	6	Specify study characteristics (e.g., PICOS, length of follow-up) and report characteristics (e.g., years considered, language, publication status) used as criteria for eligibility, giving rationale.	Page 5
Information sources	7	Describe all information sources (e.g., databases with dates of coverage, contact with study authors to identify additional studies) in the search and date last searched.	Page 5, 6
Search	8	Present full electronic search strategy for at least one database, including any limits used, such that it could be repeated.	Page 4
Study selection	9	State the process for selecting studies (i.e., screening, eligibility, included in systematic review, and, if applicable, included in the meta-analysis).	Page 5
Data collection process	10	Describe method of data extraction from reports (e.g., piloted forms, independently, in duplicate) and any processes for obtaining and confirming data from investigators.	Page 5

Data items	11	List and define all variables for which data were sought (e.g., PICOS, funding sources) and any assumptions and simplifications made.	Page 5
Risk of bias in individual studies	12	Describe methods used for assessing risk of bias of individual studies (including specification of whether this was done at the study or outcome level), and how this information is to be used in any data synthesis.	Page 6
Summary measures	13	State the principal summary measures (e.g., risk ratio, difference in means).	Page 6
Synthesis of results	14	Describe the methods of handling data and combining results of studies, if done, including measures of consistency (e.g., I^2) for each meta-analysis.	Page 6

**Section/topic**	**#**	**Checklist item**	**Reported on page #**
Risk of bias across studies	15	Specify any assessment of risk of bias that may affect the cumulative evidence (e.g., publication bias, selective reporting within studies).	Page 6
Additional analyses	16	Describe methods of additional analyses (e.g., sensitivity or subgroup analyses, meta-regression), if done, indicating which were pre-specified.	Page 6
**RESULTS**			
Study selection	17	Give numbers of studies screened, assessed for eligibility, and included in the review, with reasons for exclusions at each stage, ideally with a flow diagram.	Page 7
Study characteristics	18	For each study, present characteristics for which data were extracted (e.g., study size, PICOS, follow-up period) and provide the citations.	Page 7
Risk of bias within studies	19	Present data on risk of bias of each study and, if available, any outcome level assessment (see item 12).	Page 7
Results of individual studies	20	For all outcomes considered (benefits or harms), present, for each study: (a) simple summary data for each intervention group (b) effect estimates and confidence intervals, ideally with a forest plot.	Page 7-10
Synthesis of results	21	Present results of each meta-analysis done, including confidence intervals and measures of consistency.	Page 7-10
Risk of bias across studies	22	Present results of any assessment of risk of bias across studies (see Item 15).	Page 10
Additional analysis	23	Give results of additional analyses, if done (e.g., sensitivity or subgroup analyses, meta-regression [see Item 16]).	Page 9, 10

**DISCUSSION**			
Summary of evidence	24	Summarize the main findings including the strength of evidence for each main outcome; consider their relevance to key groups (e.g., healthcare providers, users, and policy makers).	Page 10
Limitations	25	Discuss limitations at study and outcome level (e.g., risk of bias), and at review-level (e.g., incomplete retrieval of identified research, reporting bias).	Page 13
Conclusions	26	Provide a general interpretation of the results in the context of other evidence, and implications for future research.	Page 13
**FUNDING**			
Funding	27	Describe sources of funding for the systematic review and other support (e.g., supply of data); role of funders for the systematic review.	Page 13

From: Moher D, Liberati A, Tetzlaff J, Altman DG, The PRISMA Group (2009). Preferred Reporting Items for Systematic Reviews and Meta-Analyses: The PRISMA Statement. PLoS Med 6(6): e1000097. doi:10.1371/journal.pmed1000097

For more information, visit: **www.prisma-statement.org**.
